# The multiple maternal legacy of the Late Iron Age group of Urville-Nacqueville (France, Normandy) documents a long-standing genetic contact zone in northwestern France

**DOI:** 10.1371/journal.pone.0207459

**Published:** 2018-12-06

**Authors:** Claire-Elise Fischer, Anthony Lefort, Marie-Hélène Pemonge, Christine Couture-Veschambre, Stéphane Rottier, Marie-France Deguilloux

**Affiliations:** 1 De la Préhistoire à l’Actuel, Culture, Environnement, Anthropologie–UMR 5199, CNRS, Université de Bordeaux, Allée Geoffroy Saint-Hilaire, CS, Pessac Cedex, France; 2 Inrap Grand-Ouest, Boulevard de l’Europe, Bourguébus, France; Universitat Pompeu Fabra, SPAIN

## Abstract

The compilation of archaeological and genetic data for ancient European human groups has provided persuasive evidence for a complex series of migrations, population replacements and admixture until the Bronze Age. If the Bronze-to-Iron Age transition has been well documented archaeologically, ancient DNA (aDNA) remains rare for the latter period and does not precisely reflect the genetic diversity of European Celtic groups. In order to document the evolution of European communities, we analysed 45 individuals from the Late Iron Age (La Tène) Urville-Nacqueville necropolis in northwestern France, a region recognized as a major cultural contact zone between groups from both sides of the Channel. The characterization of 37 HVS-I mitochondrial sequences and 40 haplogroups provided the largest maternal gene pool yet recovered for the European Iron Age. First, descriptive analyses allowed us to demonstrate the presence of substantial amounts of steppe-related mitochondrial ancestry in the community, which is consistent with the expansion of Bell Beaker groups bearing an important steppe legacy in northwestern Europe at approximately 2500 BC. Second, maternal genetic affinities highlighted with Bronze Age groups from Great Britain and the Iberian Peninsula regions tends to support the idea that the continuous cultural exchanges documented archaeologically across the Channel and along the Atlantic coast (during and after the Bronze Age period) were accompanied by significant gene flow. Lastly, our results suggest a maternal genetic continuity between Bronze Age and Iron Age groups that would argue in favour of a cultural transition linked to progressive local economic changes rather than to a massive influx of allochthone groups. The palaeogenetic data gathered for the Urville-Nacqueville group constitute an important step in the biological characterization of European Iron age groups. Clearly, more numerous and diachronic aDNA data are needed to fully understand the complex relationship between the cultural and biological evolution of groups from the period.

## Introduction

The confrontation between the complementary archaeological and ancient DNA (aDNA) arguments provides invaluable insights into the role of biological processes in major cultural changes identified in the archaeological record. Multidisciplinary approaches test the potential correlation between (supra-)regional cultural groups and genetically differentiable individuals, or to what extent the cultural hiatuses documented for diverse periods were linked to human population turnover. The compilation of such combined data for European human groups from the Palaeolithic to the Bronze Age period have provided persuasive evidence for a complex series of expansions, population replacements and resurgences, and admixture between divergent groups (see Harris 2017 [1] for a recent review). This complex biological evolution resulted in the fact that the great majority of European populations’ ancestry derived from three distinct sources: (i) a “hunter-gatherer”-related ancestry inherited mainly from Mesolithic human groups, (ii) a “Neolithic farmer”-related ancestry linked to the migration of farmers originating from northwestern Anatolia and linked to the Neolithic expansion into Europe, and (iii) a “steppe-related”-ancestry reflecting the expansion into Europe during the third millennium BE of pastoralist groups originating from the Pontic-Caspian steppes and presenting genetic affinities with individuals associated with the Yamnaya complex [2–4]. This last steppe-related ancestry was disseminated into Europe through the expansion of two cultural complexes, the Corded Ware and Bell Beaker complexes that overlapped geographically during the Early Bronze Age [2,3,5–7] (S1A Fig). Indeed, palaeogenomic studies focusing on the Late Neolithic-to-Bronze Age transition have shown that up to 75% of the ancestry of individuals associated with the Corded Ware and Bell Beaker complexes in northern and northwestern Europe could be traced to populations originating from the steppes [2,3]. Thus, current data indicates massive migration from the steppes, rather than a constant gene flow, was responsible for this impressive genetic turnover in the European populations concerned [3]. This major genetic transformation is also detected through the evolution of uniparental markers (mitochondrial DNA -mtDNA- and Y-chromosome), with Neolithic haplogroups being replaced by new maternal (I, T1, U2, U4, U5a, W and subtypes of H) and paternal (R1a and R1b) lineages originating from eastern regions [2,3].

In the context of the evolution of European populations, the Beaker complex holds a special and more complex status than older archaeological complexes, since it does not correspond to a genetically homogeneous population [6]. Recently published genome-wide data have, in fact, provided persuasive arguments in favour of the combination of two distinct processes—cultural transmission and human migration—in different regions being responsible for the expansion of the Beaker cultural complex. These recent studies also showed that Bell Beaker groups in Britain were genetically distinct from Neolithic populations from the same region and therefore had migrated from the continent, while in Iberia, indications of genetic continuity between these same groups consolidated the idea of cultural transmission with more northern groups [6]. The small-scale influence of migration into the Iberian peninsula led to the very low steppe-related ancestry in the region’s Bronze Age group when compared to Britain [6,7].

The subsequent major transition in Europe occurred during the last millennium BE and corresponded to the emergence of the Iron Age. If this transition is archaeologically well documented, only rare aDNA data are currently available for the period to characterize the biological diversity of European Celtic groups with any degree of precision [8–12]. Iron use was documented in Western Europe during the eighth century BE and shortly after for the British Isles and Northern Europe. Different hypotheses have been put forward to explain the expansion of iron use across Europe. The “migration hypothesis” proposed a new and major migration of groups originating from Eastern Europe in the cultural diffusion. This hypothesis found support in linguistic studies and in the adoption, in Gaul in particular, of the Hallstattian sword originating from Central Europe [13]. The second “evolutionary hypothesis” was based on the perception of progressive economic changes and instead proposed a cultural and biological continuity between the Bronze and Iron Age periods [13].

In terms of ancient French groups, aDNA data is rare for the Late Neolithic (N = 4 in [6]), whereas no aDNA data have so far been published for the Bronze and Iron Age. Consequently, our study aims to provide new insights into the genetic legacy of these ancient groups through the palaeogenetic analysis of the Late Iron Age (La Tène D1 period [14]) cemetery at Urville-Nacqueville in northwestern France (Normandy region; S1 Fig). The northwestern French regions, and more particularly Normandy, held a special position in the regional archaeological context given a period of long cultural exchange with groups from southern Britain. Cultural exchanges across the Channel became particularly evident from the Bell Beaker and persisted throughout the Bronze Age period [15]. Convincing evidence of these exchanges is found in various shipwrecks documented in the Channel, such the Dover boat, dating from about 1550 BE [16]. During the Early Bronze Age, competition between contemporaneous continental “tumulus Armoricains” and Wessex mounds in south central England illustrated the need to express the prestige of the elites who presumably controlled long-distance trade in raw materials and prestige goods (gold, lignite, amber, copper, etc.) across the Channel. Exchanges between the continent and the British Isles persisted and intensified during the Middle and Late Bronze Ages, leading to the formation of a broad cultural entity common to both sides of the Channel: the “Manche—Mer du Nord (MMN) complex” [17,18] (S1B Fig). Exchanges likely persisted on a smaller scale during the Iron Age, even if their intensity was difficult to demonstrate archaeologically before the Late Iron Age, when many new continental imports reached southern Britain (Mediterranean imports, pottery and coins) [19,20]. Lastly, as the Roman provinces expanded, textual sources were found to frequently evoke numerous resources originating from northwestern France and southern Britain. In *De Bello Gallico*, written by Julius Caesar between 58 and 50 BE, numerous passages attested more or less explicitly to the intensity of exchanges linking northern Gaul and Britain. In short, the different sources available did not present the Channel as a barrier but rather as a particular, dynamic maritime axis during the Bronze and Iron Age periods. Consequently, the Channel shores grouped a wide range of maritime sites devoted to military, commercial or fishing activities [19–23].

**Fig 1:**
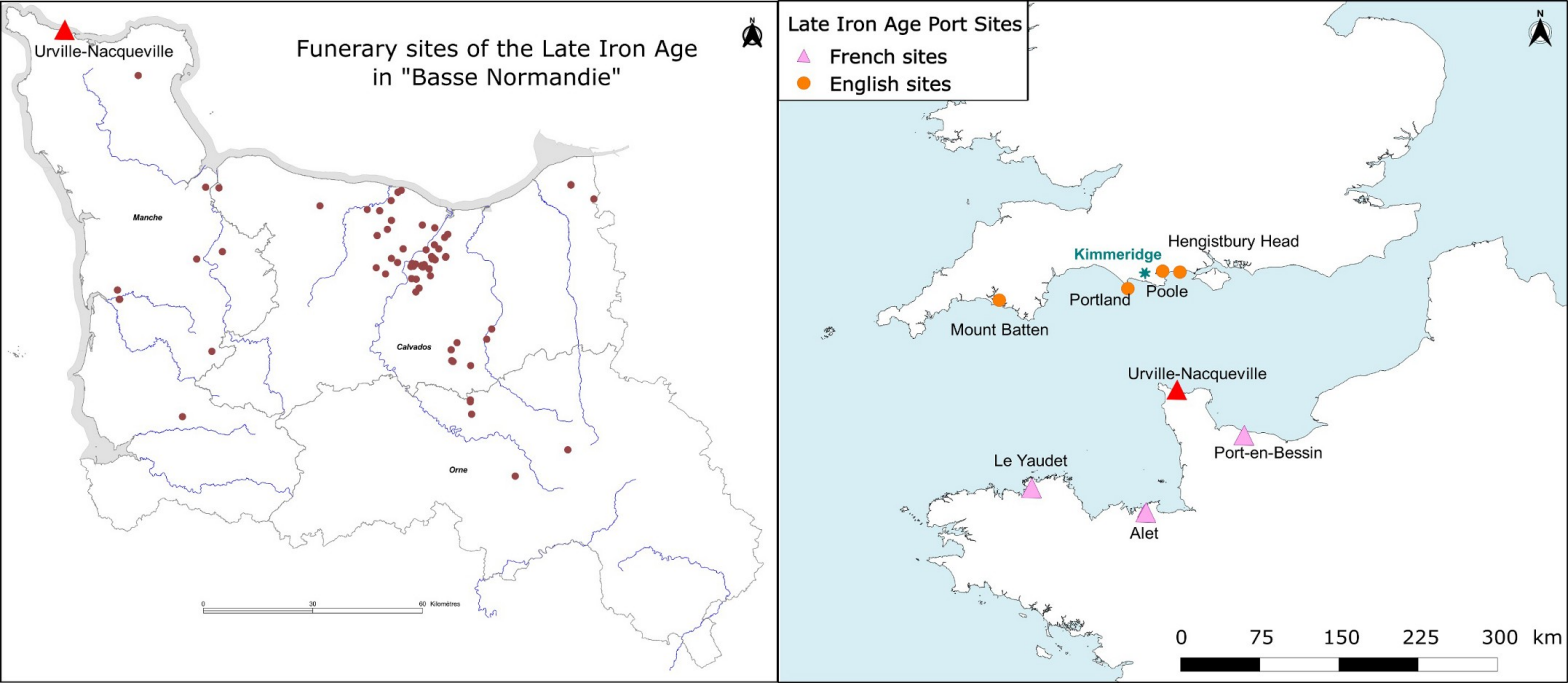
Location of Urville-Nacqueville. **A)** with Late Iron Age necropolises in Lower Normandy and **B)** with Late Iron Age port sites along the Channel.

In this complex interaction context, the Urville-Nacqueville site (hereafter named “UN”) holds a key geographical position. Located near Cherbourg at the middle of the French Channel coast, it was only approximately 60 nautical miles (around 110 km) from the English coast (south of Hengistbury Head and Poole Harbour) (Fig 1). Occupied between 120 and 80 BE, the location was one of the numerous trading posts set up for commercial exchanges between the British Isles and the mainland during Late Iron Age [24,25]. Two distinct sectors at the UN site correspond to an open settlement and a cemetery. The archaeological material collected from the settlement sector has provided firm evidence of cultural links with the Dorset coast located on the opposite Channel shore, including the Kimmeridge shale industry, British-tradition circular buildings, British pottery shards, chalk used to produce lime milk, tin ingot, or raw lead granules used for bronze working [24,25]. The second sector corresponds to an important cemetery that is currently unique in Normandy for the Late Iron Age (Fig 1). Its uniqueness lies not only in the important number of burials recovered (more than one hundred individuals [26]) compared to other contemporary funerary sites in the region grouping generally less than 40 burials [27] but this cemetery also combines several types of body treatments (*i*.*e*., cremation and burials [26]), and cremation was revealed to be almost exclusive to this local archaeological context [27]. Lastly, the discovery of some individuals with a more or less pronounced flexion of the lower limbs is reminiscent of the position observed in the Durotrigan burials described at contemporary sites in the Dorset region [28]. Consequently, the chronological and geographical characteristics of the UN necropolis, combined with the important number of human remains discovered, offers a unique opportunity to document the legacy and genetic interactions of a Late Iron age community from Western Europe. A key question in our study of the uniparental markers (mtDNA and Y chromosome) within the UN community was to characterize the maternal and paternal genetic legacy of the group by comparing its gene pool to those of ancient or contemporary groups for which genetic data were available. A related question was whether the genetic affinities discovered between the Late Iron Age UN group and preceding Bronze Age groups could enable us to determine if the cultural transition between the Bronze and Iron Age periods was accompanied by human population turnover. Another question was whether the cultural exchanges document in the archaeological between the British Isles and the continent were accompanied by significant gene flow. Finally, we tested for potential genetic continuity between the Late Iron Age UN group and extant European populations. In this context, it is important to note that, in keeping in line with multiple political needs, the Gauls (Celts from the French territory) have for some time been presented as the primary ancestors of actual French populations. Since the end of the nineteenth century and following military defeats against the German, the Gauls have been raised to the rank of the original French, and the idea of “Our ancestors the Gauls” can still be found in history books and scholarly studies. Consequently, our study, which documents for the first time the genetic legacy of these ancient groups, aims to participate in the ongoing re-examination of this Gallic status and identity in the context of French history [29].

## Material and methods

### Archaeological samples

A total of 94 graves were discovered in the Urville-Nacqueville necropolis (64 burials, 28 cremations and 2 mixed) containing the remains of at least 108 individuals. The cremations were distributed in the southwest sector of the necropolis, whereas burials were organized inside (83%) or outside (17%) a square enclosure of approximately 16 metres on each side. All aDNA analyses were conducted on individuals recovered from burials (i.e. not cremated). Genetic analyses targeted 45 individuals distributed across the entire necropolis, inside and outside the enclosure, to explore the genetic composition of the UN population as a whole (S1 Table and S2 Fig). The human remains selected for the study originated either from individual and isolated burials, overlapping burials, grouped burials or represented isolated remains (such as adult mandible 83-bis found near child grave 83). No selection was made according to age, meaning that the overall sample included 24 individuals from the foetal stage up to 1 year old, 11 children aged 1–7 years, 1 adolescent aged 10–12 years, 4 young adults aged 15–23 years, 4 adults and 1 adult over 30 years (biological analyses were conducted by S. Rottier) [14]. Based on the available remains, we sampled (in order of preference) petrous bone, teeth or various bones presenting good macroscopic conservation (S1 Table). Human remains were almost all sampled during excavations, with all necessary precautions taken to limit contamination. Only rare individuals were re-sampled in the lab, long after excavations and biological analyses, to replicate several problematic genetic profiles. Since these samples had been significantly handled, we followed a very strict decontamination protocol (see below), including genotyping all individuals who had come in contact the material. All established aDNA guidelines were then followed to minimize contamination during all subsequent steps of analyses conducted in the aDNA facilities of the UMR PACEA (Bordeaux University) (44.80; -0.61).

### Ancient DNA extraction

The samples were systematically submitted to a treatment of bleach and UV radiation, reduced to powder, and incubated overnight in lysis buffer (0.5 M EDTA, pH = 8, 25 mg/mL proteinase K and 0.5% N-Lauryl sarkosyl). Since the DNA isolation procedure evolved during the course of the study, DNA from 23 individuals was extracted using the purification kit NucleoSpin Extract II (Macherey-Nagel, Düren, Germany), whereas DNA from 45 individuals or replicates were extracted using the MinElute purification kit (Qiagen). For the second set of DNA isolation using the MinElute kit, bone powder or teeth fragments were submitted to a bleach wash as described in [30]. Lastly, since the first analyses carried out on DNA extracts showed clear inhibition indices (null SNPs profiles), an additional purification step was conducted using the PowerClean kit (MoBio).

### aDNA analyses

Eighteen mitochondrial and ten Y chromosome SNPs (Single Nucleotide Polymorphisms) enabling the characterization of primary ancient European maternal and paternal lineages were typed through one multiplex using MALDI-TOF MS-based SNP genotyping (iPLEX Gold technology, Sequenom, Inc, San Diego, CA). All primers used for these experiments and procedure details are available in Rivollat *et al*. [31]. To determine the maternal haplotype of each individual, four overlapping fragments of the HVS-1 region of the mtDNA (nps 16,024±16,380) were amplified and analysed, following the procedures described in Rivollat *et al*. [31]. Moreover, to trace potential contaminations, all individuals who manipulated the samples were genotyped (S2 Table).

All results (SNPs typing and HVS-1 fragment amplifications) were replicated on at least two different extracts per individual, and the lineages obtained were systematically compared to the those characterized for persons having manipulated the samples. As a consequence, the HVS-I consensus sequences retained for subsequent analyses were those (i) presenting concordant results between distinct replications, (ii) enabling coherent lineage attribution through SNPs and HVS-I typing, and (iii) presenting mtDNA haplotypes distinct from the manipulators. All reported mutations were established according to the revised Cambridge Reference Sequence (rCRS) [32,33]. Each individual’s haplogroup and haplotype was determined based on PhyloTree.org (mtDNA tree build 17) [34].

### Descriptive analyses

To assess the genetic affinities between the Urville-Nacqueville community and other ancient populations, we compiled a total of 1433 published mtDNA data sequences dating from the Palaeolithic to the Iron Age periods (42,500–300 cal. BE) (Beau *et al*. (2017) [35] dataset actualised for more recent periods; S3 Table). The global sequence dataset was divided into 29 chronological, geographical and cultural groups to discuss (i) the implication of different anterior groups in the UN gene pool constitution and (ii) the relationships of the UN group with contemporaneous populations. To document the genetic legacy shared between the UN group and current European populations, we used 16,901 modern HVS-1 sequences (nps 16,024–16,380) originating from 34 modern populations distributed over the continent (database from Rivollat *et al*. 2015 [31]; S9 Table). Since important regional variability between maternal gene pools from distinct French regions has been demonstrated [36], we independently considered distinct French regional groups to unravel potential regional variation affinities with the UN community.

Population genetic diversity parameters, proportion of shared mtDNA haplotypes and population-specific pairwise genetic distances (F_ST_) were computed using the Arlequin software (version 3.5.1.2) [37]. A classic principal component analysis (PCA) of mtDNA haplogroups frequencies was carried out, taking into account the major identified European haplogroups and distinguished in aDNA literature targeting European populations: H, HV, I, J, K, N, T, U4, U5, U, V, W, X and “others”. The PCA was performed using RStudio (version 1.0.153) and the FactoMineR package.

## Results and discussion

### Urville-Nacqueville genetic diversity

Among the 45 individuals analysed, 39 exploitable mitochondrial SNP profiles were obtained (87% success rate), enabling a French Iron Age group maternal gene pool to be characterised for the first time. In contrast, only four individuals provided partial profiles for the Y chromosome SNPs, suggesting, as is usually the case for aDNA, a more difficult access to nuclear DNA at the UN site (S4 Table). More powerful palaeogenomic analyses, enabling better access to nuclear genomes than classical PCR-based methods, may provide more fruitful paternal lineage characterization. Y-chromosome detection had some inconsistencies, but pointed to the presence of R haplogroups for three individuals. Further differentiation between sub-haplogroups R1a/R1b was not possible since attempts to identify R sub-lineages produced conflicting results. Whatever the case, due to low documented Y chromosome diversity, subsequent analyses relied on the maternal gene pool characterized for the UN group. Age-dependent funeral treatment has been proposed for the UN necropolis. In this respect, individuals under the age of 10 were buried while older individuals were cremated [38]. Consequently, we considered that the mitochondrial gene pool identified for buried individuals was representative, at least in part, of overall gene pool for the entire population.

From the individuals providing mtDNA data, 37 complete and authenticated HVS-1 sequences (or haplotypes; see details in S1 Table) could be characterized. These results showed an excellent conservation of mitochondrial DNA from the UN individuals (for 82.2% of them). These results may appear counter-intuitive, since the UN necropolis was found in a unique and humid context, *i*.*e*., on the beach along the Channel coast. However, these environmental conditions, including repeated submersion due to sea level fluctuations, appear not only to have created exceptional preservation conditions for DNA but also for diverse organic materials such as wooden objects or unidentified organic substances along the bones [25]. Nevertheless, it is important to note that an additional purification step (with a kit developed for DNA containing high amounts of humic substances, polyphenolics, polysaccharides and other PCR inhibitors) was necessary to recover sufficiently pure DNA for downstream analyses from this unique archaeological context.

To the best of our knowledge, the mtDNA gene pool characterized for the UN community is the largest ever recovered for a European Iron Age context. Although subsequent descriptive analyses were based only on maternal lineages, such an important mtDNA dataset for one ancient community enables us to discuss group affinities with a high degree of confidence. A noticeable variety of mtDNA haplogroups characterized the UN group, including K1, J1, H, H1, H2, H3, H5, H6/8, H11, U4, U5, I, V, T1 and T2 major lineages (S2 Fig). A total of 14 different sub-haplogroups and 23 distinct haplotypes could be determined. Since seven distinct mtDNA haplotypes were shared by at least two individuals, we anticipated that some UN individuals might be closely maternally related (S1 Table). The impact of these potential kinships within the necropolis of Urville-Nacqueville on different downstream analyses was verified and proved to have no significant effect on the results (see details below).

The genetic diversity of the Urville-Nacqueville community (Hd = 0.9610 +/- 0.005996) was found to be similar to the diversity values measured for diverse Neolithic, Chalcolithic or Bronze Age groups (S5 Table). Compared to other Iron Age groups, the Urville-Nacqueville’s genetic diversity is slightly inferior to the values obtained for Scythian groups from the Black Sea region (Hd = 0.9942 +/- 0.0193), whereas it is higher than the values obtained for Iron Age groups from Germany and Spain (Hd = 0.9167 +/- 0.0920 and Hd = 0.8063 +/- 0.0785 respectively). However, the low diversities measured for these two latter groups could be the result of the small number of individuals investigated. It is worth noting that similar maternal diversity could be determined for ancient groups geographically and chronologically widely distributed (for example the EN_MN_Germany_Alsace group, which includes 137 individuals originating from Germany and Alsace and dating from 5500 to 4000 cal. BE) and for a Gallic community who used the Urville-Nacqueville necropolis for approximately 150 years. The important mtDNA diversity characterized for the UN group may have resulted from two exclusive or combined processes. First, the UN site has been described as a port that witnessed intensive exchanges with Great Britain. The documented cultural exchanges may have been correlated to high group mobility and the arrival (and burial) at UN of individuals originating from various locations. Second, an important mobility of female group members may be linked to a patrilocal matrimonial system where women migrated to join their husbands’ group. This type of matrimonial system has already been genetically demonstrated for earlier periods, such as the Neolithic and the Bronze Age [39–41]. For the Iron Age, studies relying on textual sources (Greek and Roman texts) would support such a matrimonial system for societies considered patriarchal and patrilineal [42]. Unfortunately, the absence of sufficient Y chromosome data in the present study did not allow us to directly test this second hypothesis.

### The maternal genetic legacy of the Urville-Nacqueville community

**Fig 2:**
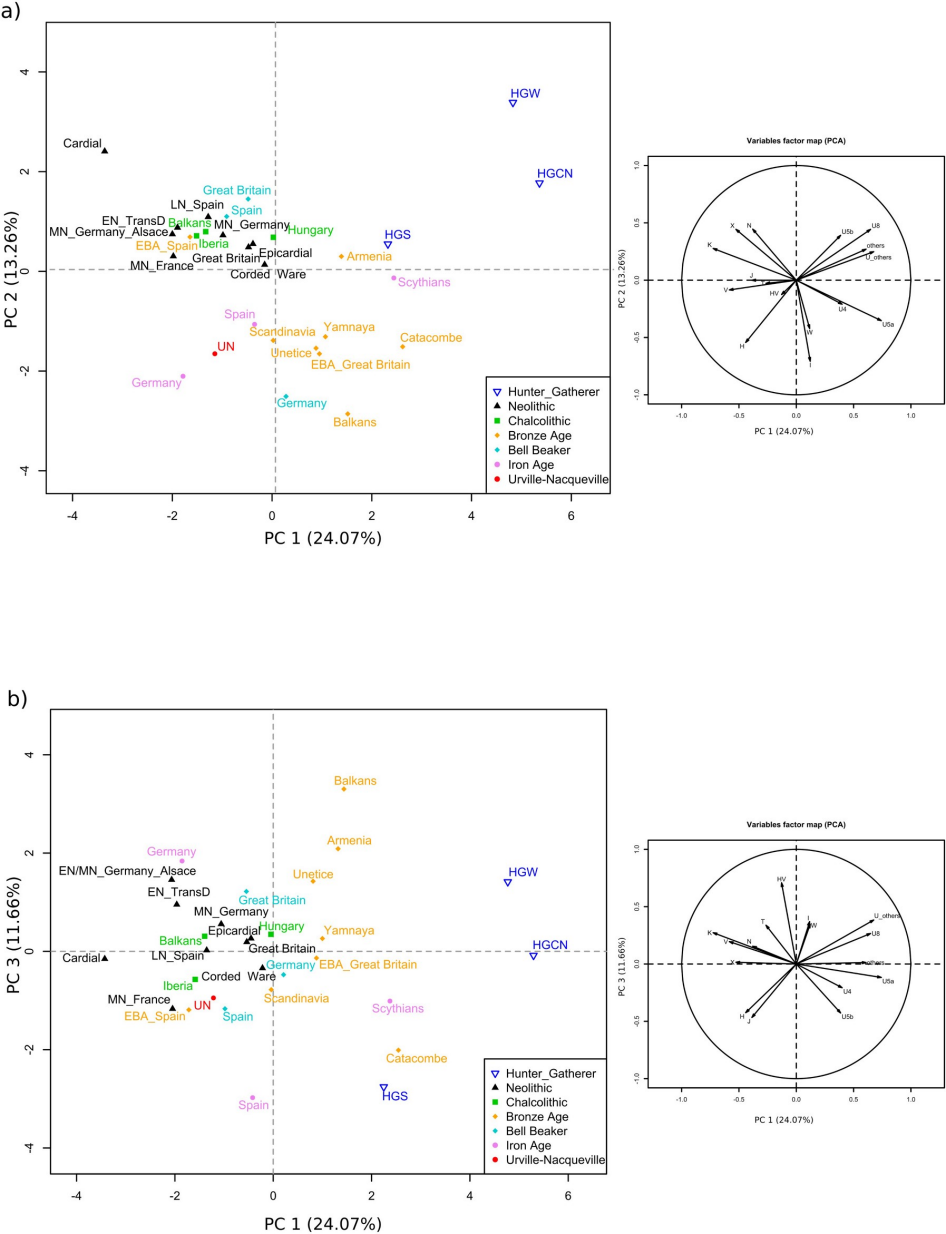
Principal component analysis (PCA) on the ancient mtDNA dataset. **A)** PCA performed on haplogroup frequencies, built with PC1 and PC2 **B)** PCA performed on haplogroup frequencies, built with PC1 and PC3. HGCN: Hunter-gatherers from Central Europe; HGW: Hunter-gatherers from Western Europe; HGS: Hunter-gatherers from Southern Europe; CAR: Cardial; EN: Early Neolithic; MN: Middle Neolithic; LN: Late Neolithic; UN: Urville-Nacqueville.

For the principal component analyses (PCA), we merged the mtDNA haplogroup frequencies characterized for the UN community with the frequencies published for ancient groups. Since potential maternal kinship within the Urville-Nacqueville necropolis had previously been noted (which may bias haplogroup frequency estimations), we carried out PCA either considering all the UN individuals (Fig 2) or only one individual per haplotype shared between several individuals (see S3 Fig). As the exclusion of potentially maternally related individuals had no significant effect on the PCA structure, we decided in the main text to present the PCA obtained for the entire UN population.

The PCA constructed with PC1 and PC2 (Fig 2B) revealed that most of the variations in mtDNA haplogroup frequencies lie between ancient European hunter-gatherers, Neolithic and Bronze Age groups. In this PCA, the Urville-Nacqueville group is positioned near other Iron Age groups from Spain and Germany, whereas all Iron Age groups appeared close to the European Bronze Age cluster. The UN position in the PCA was influenced by both (i) a noticeable proportion of haplogroups U4 and I, a characteristic shared with European Bronze Age groups who derived most of their ancestry from populations related to Early Bronze Age Yamnaya pastoralists [2,3] that lowered groups along the y-axis, and (ii) by frequencies of haplogroups V and U5 that differentiated the UN group from Bronze Age populations (along the x-axis). The second PCA, built with PC1 and PC3 (Fig 2A), tended to associate different chronological groups with a common European region, suggesting noticeable regional maternal continuity. According to PC1 and PC3, German and British groups dated to Early Bronze Age or associated with the Bell Beaker complex clustered near the Yamnaya group, thus providing evidence for a strong heritage of steppe groups. In this second PCA, the UN community appeared close to Chalcolithic to Early Bronze Age groups from Spain. Urville-Nacqueville's position near the French Middle Neolithic sample (MN_France) indicates a possible regional continuity in France.

The first important outcome was then a clear maternal steppe legacy in the UN community, which is supported by several distinct lines of evidence. In this respect, the PCA analysis demonstrated clear maternal affinities between the UN group and European Bronze Age groups bearing an important steppe legacy. Indeed, the UN community was characterized by a noticeable frequency of mtDNA haplogroup U4 (5.13%) similar to those found in Yamnaya pastoralists (YAM 6.66%) or groups presenting a major steppe-ancestry component: 6.89% for groups linked to Corded Ware (CWC), 4.91% for groups associated with Bell Beaker in Germany (BBC_GER) or 4.54% for Early Bronze Age groups from Great Britain (EBA_GB) (S6 Table). Similarly, the UN group shared significant frequency of haplogroup I (5.12%) with Yamnaya pastoralists (YAM 2.22%), groups from the Corded Ware context (CWC 1.72%), groups associated with Bell Beaker in Germany (BBC_GER 3.27%) or even Early Bronze Age groups from Great Britain (EBA_GB 13.6%). The sharing of several very specific mtDNA haplotypes between the UN group and ancient groups with steppe-related ancestry corroborated these observations. It is therefore worth noting that the H6/8_16362 haplotype is shared between the UN and Yamnaya (YAM), Corded Ware (CWC), and Bell Beaker groups from Great Britain (BBC_GB); the T1a_16126-16163-16186-16294 haplotype between UN and Yamnaya (YAM), Corded Ware (CWC) and Bell Beaker groups from Germany (BBC_GER); the H11_16293–16311 between UN and Bell Beaker groups from Germany (BBC_GER); and, lastly, the sharing of the I_16129-16223-16391 haplotype between UN and groups associated with Corded Ware (CWC) or the Early Bronze Age from Great Britain (EBA_GB) (S7 Table). We then considered the frequencies of shared informative haplotypes between UN and other ancient groups. By “informative haplotypes”, we mean haplotypes that are not ubiquitous among ancient groups and hence provide phylogeographic information. Affinities could then be highlighted with the Yamnaya pastoralists (YAM, 14.6% shared informative haplotypes) and groups bearing an important steppe legacy: the Catacomb group (CAT, 45.8%), groups associated with the Corded Ware complex (CWC, 22%), Early Bronze Age groups from Great Britain (EBA_GB, 18.2%), the Unetice group (UNE, 16.7%), and Bell Beaker groups from Germany (BBC_GER, 14.8%) (S7 Table). In addition, the F_ST_ measured between ancient groups revealed only a small genetic distance between the Urville-Nacqueville group and ancient groups associated with the Corded Ware complex (CWC) or Bell Beaker (BBC_GB) and Early Bronze Age groups from Great Britain (EBA_GB) (F_ST_ values of 0.005, 0.00514 and 0.00238 respectively) (S8 Table and S4 Fig). Lastly, the four UN individuals that yielded partial data for Y-chromosome SNPs carry alleles diagnostic of the R haplogroup that is primarily associated with the arrival of steppe migrants in central Europe after 3000 BC (S4 Table) [3].

Previous studies have shown that the expansion of the Corded Ware complex in north-central and northeastern Europe was associated with people of whose major ancestry could be traced to a population originating from the Pontic–Caspian steppe in the third millennium BE and who presented genetic affinities with individuals from the Yamnaya complex [2,3]. This steppe legacy was transmitted to groups associated with later archaeological cultures such as the Bell Beaker complex. Recent genome-wide data have notably demonstrated that the migration of Beaker groups form Central Europe to Great Britain accompanied the expansion of the Beaker complex in the region. This migration was responsible for the introduction of high levels of steppe-related ancestry in Great Britain, leading to an important population turnover [6]. Since all lines of evidence in our study demonstrated (i) a clear steppe-related ancestry in the Iron Age group of Urville-Nacqueville, combined with (ii) significant maternal affinities with Bell Beaker groups from Germany and Great Britain, a consistent hypothesis might propose that the people associated with the northern expansion of the Bell Beaker complex to Great Britain may have reached England via northern France and the Normandy region, or that people associated with the Bell Beaker complex first entered Great Britain and then crossed the Channel to reach Normandy. Another hypothesis, exclusive or combined with the first one, would see intensive and continuous gene flow between groups from both sides of the Channel, during and after Bell Beaker period, as being responsible for the steppe-related heritage expansion from Great Britain to Normandy. Unfortunately, no genetic data is currently available for either northern French or English groups from the Late Bronze or Iron Ages to more precisely document the dynamics of genetic exchanges between both regions. Nevertheless, different archaeological arguments document intensive cultural exchanges across the Channel at least from the Bell Beaker period [43,44] lasting throughout the Bronze Age and giving rise to a cultural complex uniting both regions: the “Manche-Mer du Nord (MMN) complex” (S1B Fig). Lastly, the continuation of intensive exchanges between the Northern Gaul (including Normandy) and Southern England is attested throughout the Iron Age in the sharing of fibulae, ceramics, or architectural trends and practices (circular plan habitats [45]). The archaeological record of these continuous exchanges has led P-Y. Milcent to propose the Medio-Atlantic cultural entity (S1C Fig; [46]). Concerning more specifically the necropolis of Urville-Nacqueville the presence of what are sometimes called "Durotriges" burials, which originated in England [28], succinctly illustrates cultural influences from Great Britain. The palaeogenetic arguments provided by our study would then support important gene flow having accompanied intensive and continuous cultural exchanges across the Channel.

The second important outcome of our results was an appreciable maternal differentiation between the UN community and ancient Bronze Age groups in the PCA analysis that placed the UN group outside the Bronze Age cluster. This maternal distinction was notably linked to a lower frequency of the U5a haplogroup in the Iron Age group from France (7.69%) compared to any of the Bronze Age groups (from 11% to 20%). The maternal differentiation between the UN community and ancient Bronze Age groups is also linked to the notable presence of haplogroup V in the UN community (5.13%), whereas this haplogroup is absent or rare in European Bronze Age groups except for Early Bronze Age groups from Spain (3.57%). If the very low frequency of haplogroup V in European Bronze Age groups is potentially due to the current state of research, it is nevertheless worth noting that maternal link suggested with ancient groups from Southern Europe through similar V haplogroup frequencies was reinforced by the close genetic distances measured between the UN group and individuals associated with the Neolithic (Epicardial), Bell Beaker and Early Bronze Age complexes in Spain (F_st_ values of respectively 0.00215, 0.00288 and 0.00052) (S8 Table and S4 Fig). These observations may be linked to cultural and genetic exchanges along the Atlantic coast either during the Neolithic or Bronze Age. In fact, recent genome-wide data have provided convincing arguments showing that Neolithic populations from Great Britain were derived from migrants originating from the Iberian Peninsula and spreading along the Atlantic coast (perhaps linked to the diffusion of megalithic tombs [6]). Cultural exchanges along the Atlantic coast were also archaeologically documented during the Bell Beaker period, and persisted into the final Bronze Age, giving rise to the “Atlantic Bronze Age complex” [47,48]. The region of Normandy along the Atlantic way could have received migrants from southern regions during these different interaction episodes that left a visible mark on its Iron Age maternal gene pool. Further palaeogenetic/palaeogenomic data from geographically and chronologically distinct groups distributed along this Atlantic axis are needed to fully understand the complex dynamic interaction between these regions during the Neolithic, Bronze Age and Iron Age. Along with arguments compiled for cultural and genetic exchanges across the Channel, currently available data supports a major impact of exchanges along dynamic maritime axes on the biological evolution of ancient groups.

The last element of discussion to emerge from the descriptive analyses is a clear maternal distinctiveness of Iron Age communities originating from different regions such as France (UN), Spain (IA_Spain), Germany (IA_GER) or the Black Sea area (IA_Scy). If major differentiation is observed between the maternal gene pool characterized for European Iron Age groups *vs*. the Scythians (probably linked to Central Asian affinities of the latter), a noticeable differentiation is also evident between European Iron Age groups through their dispersion throughout the PCA and the MDS (Figs 2 and S4). Nevertheless, the maternal gene pool documented for the different Iron Age groups from Spain, Germany and the Black Sea regions may have been biased both by the small number of individuals considered (from N = 11 to N = 24; S3 Table) and the inclusion of special funerary contexts (such as the “princely seat” burial of Glauberg (Hesse, Germany; [8]) that may have been linked to the recruitment of individuals with a different status from the communities. Consequently, it appeared difficult for us to discuss the potential regional genetic differentiation of Iron Age communities based on these elements. Once again, a more precise characterization of more gene pools for Iron Age communities, distributed across the continent, is necessary to document potential genetic variations in the Celtic world. Such a genetic dataset would make it possible to test if biological differentiation existed between the Celtic cultural groups described in textual sources (Greek and Roman texts). Interestingly, the mtDNA data available for various Iron Age groups distributed across the continent suggest genetic affinities with Bronze Age groups (visible on the PCA (Fig 2)). The large maternal gene pool recovered for the Late Iron Age community of Urville-Nacqueville provided convincing arguments of maternal affinities with Bronze Age groups from Germany, Great Britain and Spain, and thus argued in favour of some genetic continuity between populations from both periods. Several authors have proposed that the transition from the Bronze to Iron Age, at least in northwestern Europe, can best be explained by progressive local economic developments that may have been linked to climatic changes (impacting agricultural practices and habitat structures) rather than by the mass influx of allochthone groups carrying new technologies [13,49]. In the same way, the subsequent development of the Late Iron Age appeared to be gradual and cumulative, linked to economic exchanges and the incursion of small populations [46]. Such a gradual cultural transformation may be responsible for local genetic continuity between groups from distinct periods and would succintly explain the persistence of the Bronze Age legacy in the Iron Age group of Urville-Nacquevile.

### Shared maternal legacy between the Urville-Nacqueville community and extant European populations

To assess potential shared maternal legacy between the Urville-Nacqueville community and modern-day populations, the maternal gene pool of the Iron Age group was compared to 16,901 HVS-1 sequences compiled from 34 extant European populations (S9 Table). Sequence data available for different French regions allows potential regional variability in terms of genetic affinity with the UN group to be explored (despite the small sample available for some regions).

**Fig 3:**
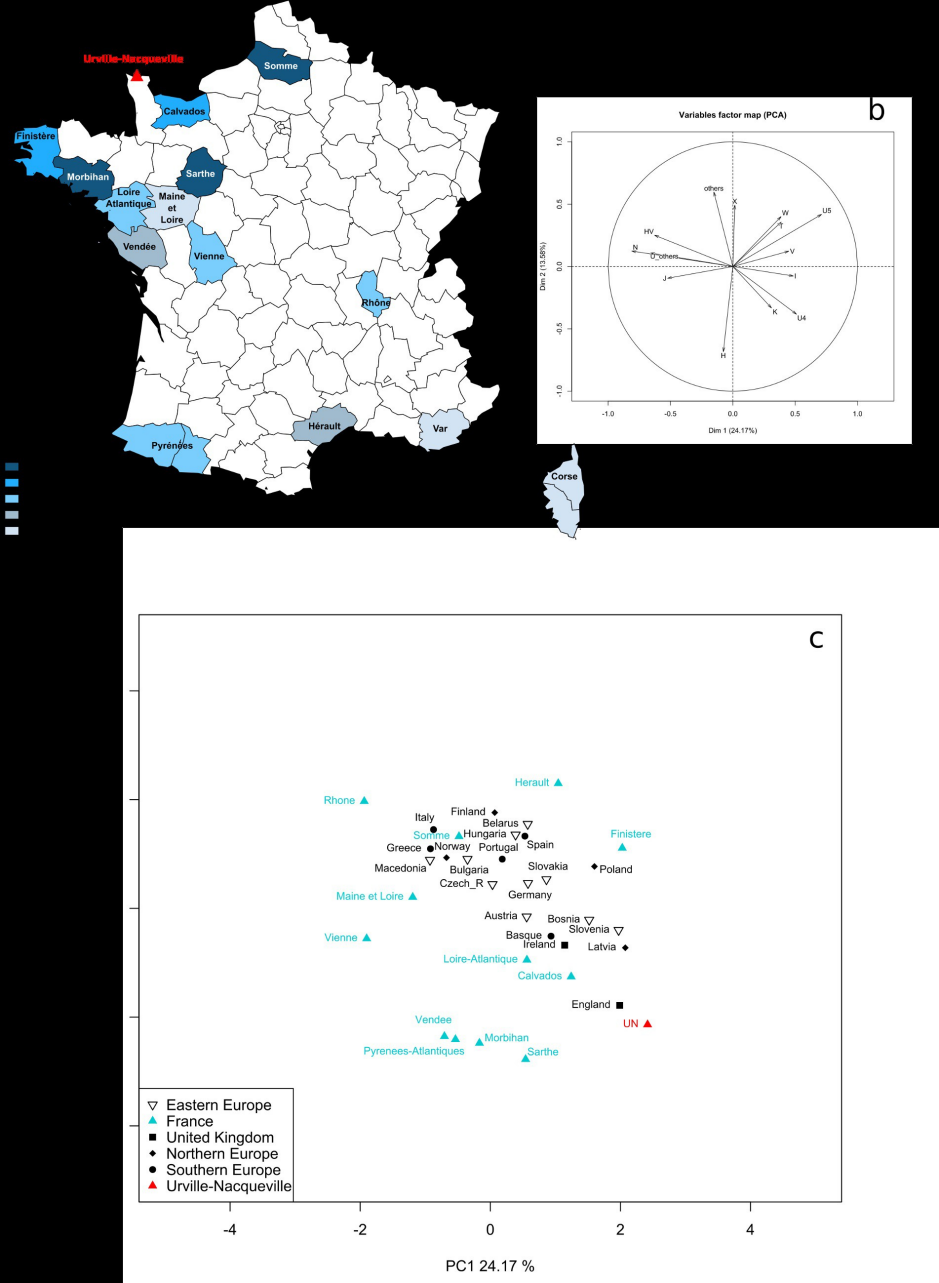
Fst and principal component analyses (PCA) for current European populations (mtDNA). **A)** Fst values measured between the UN group and extant French groups. **B)** Circle of correlation. **C)** PCA performed on haplogroup frequencies.

The PCA based on mitochondrial haplogroup frequencies (S10 Table) of extant European populations showed, on the one hand, that extant European populations were maternally homogeneous and, on the other hand, that current French regional populations were maternally heterogeneous, as already noted [36,50,51]. In this PCA, the UN ancient group falls outside the current European population cluster but close to extant populations from England and Normandy (Calvados) (Fig 3C) that represent two neighbouring regions (Fig 3A). Maternal affinities with geographically close extant populations were confirmed by the low F_ST_ values between the UN group and five extant populations from regions located in northwestern France (Sarthe, F_ST_ = 0.00211; Morbihan, F_ST_ = 0.00221; Somme, F_ST_ = 0.00385; Calvados, F_ST_ = 0.00752 and Finistere, F_ST_ = 0.00867; Fig 3A) or between UN and Irish (F_ST_ = 0.00309) or British populations (F_ST_ = 0.00338) (S11 Table). If small inconsistencies between analyses could be noticed (related to the low resolution of mtDNA data and/or the highly variable sizes of the extant populations used in the PCA), we consider that all data support a clear affinity between the UN sample and extant populations in northwestern France. Moreover, low F_ST_ values were observed between Britain and various groups in northwest France, such as Sarthe (0.00161), Calvados (0.00171) and Somme (0.00181). Interestingly, these different French regions are located, along with Britain, in the geographical area defined for the Bronze Age as the “Manche-Mer du Nord complex” (S1B Fig). These results, combined with those obtained for older groups, argue both in favour of intensive gene flow between these regions linked by maritime routes (at least since the Bronze Age period) and of regional genetic continuity between Bronze/Iron Age and extant populations, as already highlighted for Ireland [5] or Spain [9].

**Fig 4:**
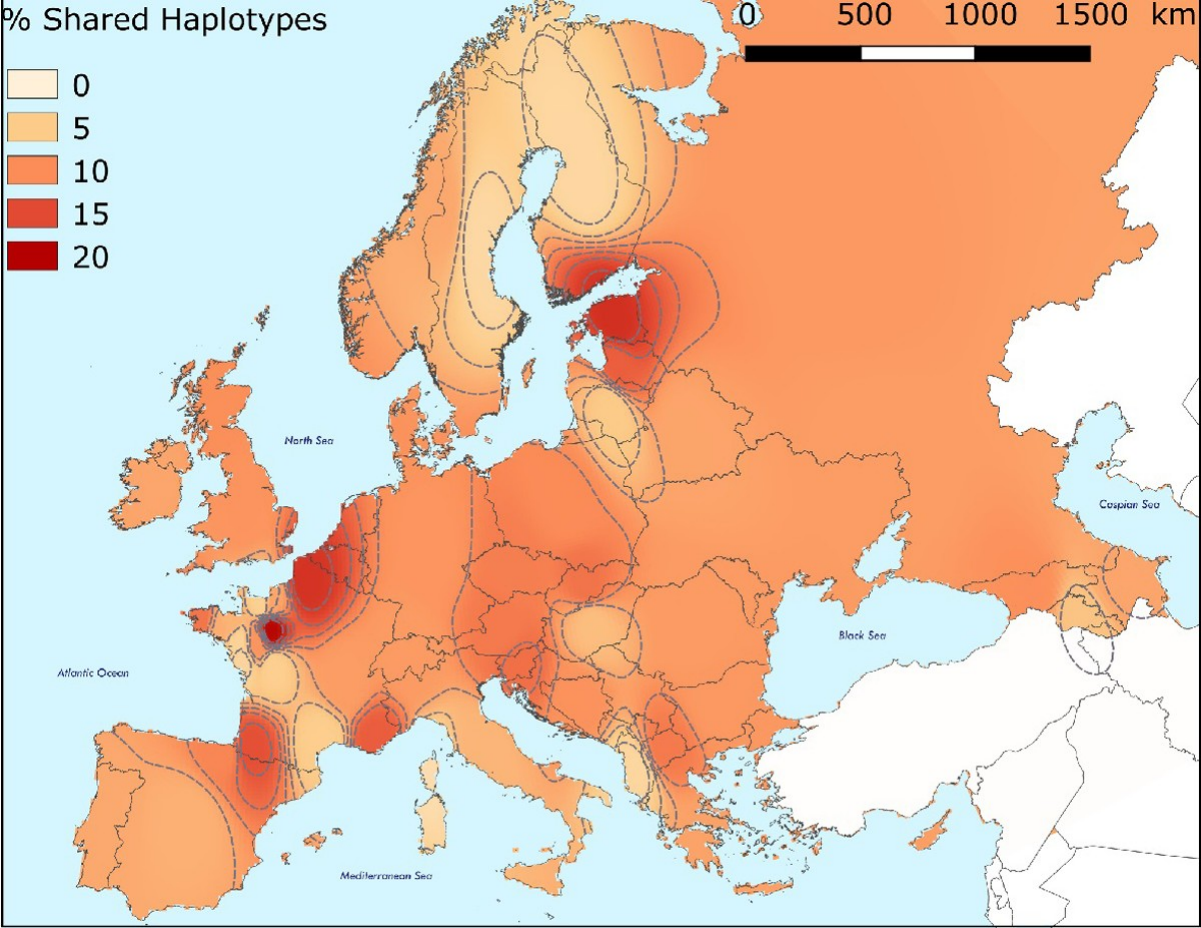
Map showing the distribution of shared haplotypes within current European populations. Map made with QGIS v.2.18.16 [54].

We then assessed the proportion of informative haplotypes shared between the ancient UN group and modern European populations to trace potential shared maternal lineage (S12 Table). Our analyses revealed three mtDNA haplotypes to be unique to the ancient UN group, since they were not found in any of the current populations considered, demonstrating the regular loss of ancient lineages during the evolution of European population. After the exclusion of ubiquitous haplotypes (mainly basal) found in a large number of European populations and then phylogeographically non-informative, the 15 remaining informative haplotypes were found to be shared between the UN group and specific extant European populations. The mapping of these shared informative haplotype frequencies enabled us to highlight interesting hotspots in northwest France, Spain, Britain, Central Europe and the Baltic region. Affinities between UN and French populations from northwestern France demonstrated by shared haplotypes further reinforces the conclusion of local maternal continuity since the Iron Age. The haplotype-sharing highlighted between the UN group and extant populations from Spain and Britain also reinforced genetic affinities already documented for ancient groups from the same regions (Bell Beaker, Early Bronze Age) and provided support for intensive and continuous genetic exchanges along the Atlantic coast. More surprisingly, the hotspots of informative haplotypes shared with the UN groups were also detected for the Baltic region (Latvia) and Central Europe (Fig 4). Among the informative shared haplotypes explaining these maternal affinities, different haplotypes affiliated to the U4, T, T1, T2, U5 and U5a haplogroups should be noted. Studies have shown that the diffusion of mtDNA haplogroups U4, T1 and U5a was mainly linked to the expansion of the pastoralist groups originating from the steppes. These haplogroups were found at high frequencies in modern Baltic populations (the Latvian population carries high frequencies of haplogroups U4 (8.8%), T (7%) and U5a1 (5.3%) [52]) and were responsible for a general trend of decreasing steppe-related ancestry in current European populations from the northeast to the southwest [3,53]. The major steppe-related ancestry preserved in extant Baltic populations is responsible for the strong maternal affinities recorded for Iron Age community from Urville-Nacqueville.

## Conclusion

Our study provides new insights into the maternal legacy of Iron Age communities in Western Europe. The description of the mtDNA gene pool of the Urville-Nacqueville community, the largest-ever characterized for a European Iron Age context, highlighted new scenarios for ancient European population interactions and evolution. The first major finding is the presence of significant amounts of maternal steppe-related ancestry in the UN community, a finding that could fit with the transmission of these lineages by both Bell Beaker groups expanding across northwestern Europe approximately 2500 BC and Bronze Age groups arriving later in France. The UN maternal diversity also proved to result from diverse and significant genetic interactions with ancient groups from Great Britain and Spain. These results are highly reminiscent of the continuous and intensive cultural exchanges documented in the archeological record on the other side of the Channel or along the Atlantic coast during and after the Bronze Age period. The palaeogenetic arguments provided by our study would therefore support an important gene flow having accompanied these maritime cultural exchanges and that the northwestern French regions may have served as a regular genetic contact zone between the north and south over extended periods. Evidently, further palaeogenetic/palaeogenomic data from different chronological groups distributed along this north-south axis are needed to more fully appreciate interaction dynamics between human groups from these regions during the Neolithic, Bronze Age and Iron Age periods.

Our study also provided convincing arguments for maternal genetic continuity both between Bronze Age groups and the UN community and between the ancient UN group and extant populations from northwestern Europe. We consider that the most parsimonious explanation for the observed genetic continuity would be a Bronze-to-Iron Age transition linked to progressive local economic changes rather than to a massive influx of allochthone groups. The second genetic continuity measured with extant populations would, to a certain extent, support an idea still found in current French scholarly books: “Our ancestors the Gauls.” Nevertheless, numerous results now available for ancient European groups have highlighted substantial population turnovers during prehistory, such as those linked to European Neolithization or the subsequent migrations of groups bearing steppe-related ancestry. Our palaeogenetic results, combined with data form the palaeogenetic / palaeogenomic literature, would argue that “our ancestors the Gauls” who actually contributed to present-day French gene pool were themselves bearers of a maternal legacy from all anterior groups, with a major contribution from late Neolithic / Bronze Age populations.

## Supporting information

S1 Fig Geographic distribution of archaeological cultures mentioned in the text. **A)** Late Neolithic / Early Bronze Age cultures, approximately 2300 BC (based on [SI.1] and [SI.2]). **B)** Late Bronze Age cultures and the “Manche-Mer du Nord complex”, approximately 1300 BC (based on [SI.3] and [SI.4]). **C)** Early Iron Age cultures and the Medio-Atlantic Iron Age, approximately 800 BC (based on [SI.5] and [SI.6]). **D)** Late Iron Age: celtophone Europe approximately 400 BC (map based on [SI.7]).
(TIFF)

S2 Fig Spatial distribution of the mitochondrial (mtDNA) haplogroups within the Urville-Nacqueville necropolis. (TIFF)

S3 Fig PCA taking into account potential kinship between UN individuals. (TIFF)

S4 Fig MDS performed on pairwise FST measured between ancient European groups. (TIFF)

S1 Table Biological and genetic data compiled for Urville-Nacqueville individuals targeted in our study. (XLSX)

S2 Table HVS-I characterised for manipulators (aDNA lab) and archaeologist (excavations or lab). (XLSX)

S3 Table Details and references of the ancient populations dataset. (XLS)

S4 Table Details of the mtDNA and Y-chromosome SNPs analysed on Urville-Nacqueville human remains. (XLSX)

S5 Table Genetic diversity indices measured on ancient groups. (XLSX)

S6 Table MtDNA haplogroups frequencies for ancient groups. (XLSX)

S7 Table Mitochondrial haplotypes shared between UN community and ancient European groups. (XLSX)

S8 Table Fst values measured between ancient European groups. (XLSX)

S9 Table Modern dataset. (XLSX)

S10 Table MtDNA haplogroups frequencies for extant European populations. (XLSX)

S11 Table Fst measured between UN and current European populations. (XLSX)

S12 Table Mitochondrial haplotypes shared between ancient UN group and extant European populations. (XLSX)

S1 File References for S1 Fig. (DOCX)
