# Correction: The multiple maternal legacy of the Late Iron Age group of Urville-Nacqueville (France, Normandy) documents a long-standing genetic contact zone in northwestern France

**DOI:** 10.1371/journal.pone.0211519

**Published:** 2019-01-25

**Authors:** The PLOS ONE staff

There are errors in the background of Fig 3. Please see the corrected Fig 3 here. The publisher apologizes for the error[s]

**Fig 3:**
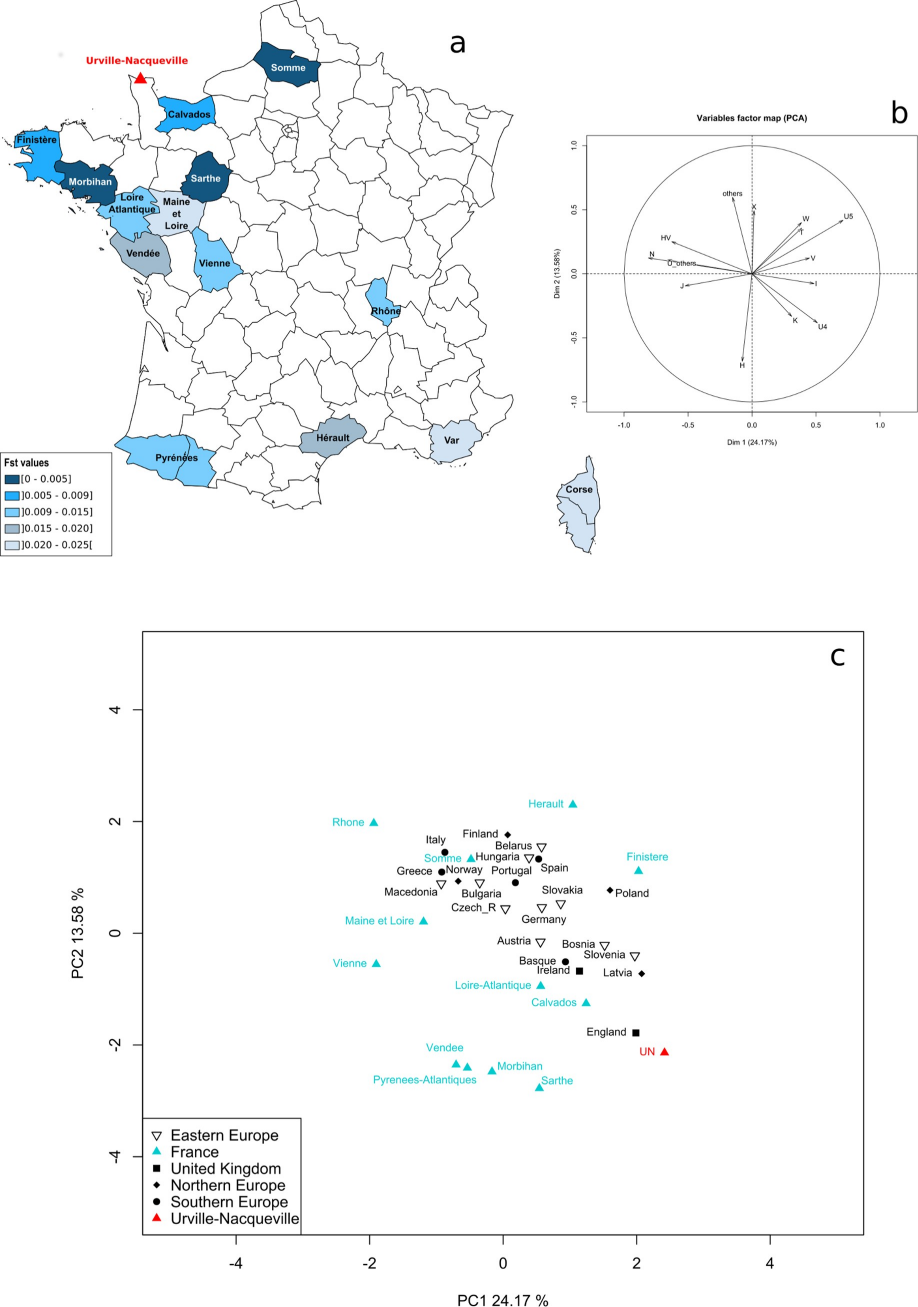
Fst and principal component analyses (PCA) for current European populations (mtDNA). **A)** Fst values measured between the UN group and extant French groups. **B)** Circle of correlation. **C)** PCA performed on haplogroup frequencies.
