# Supplementary figures and images for: The multiple maternal legacy of the Late Iron Age group of Urville-Nacqueville (France, Normandy) documents a long-standing genetic contact zone in northwestern France
Source: PLoS One. 2018 Dec 6;13(12):e0207459. doi: 10.1371/journal.pone.0207459 (PMC6283558; doi:10.1371/journal.pone.0207459)

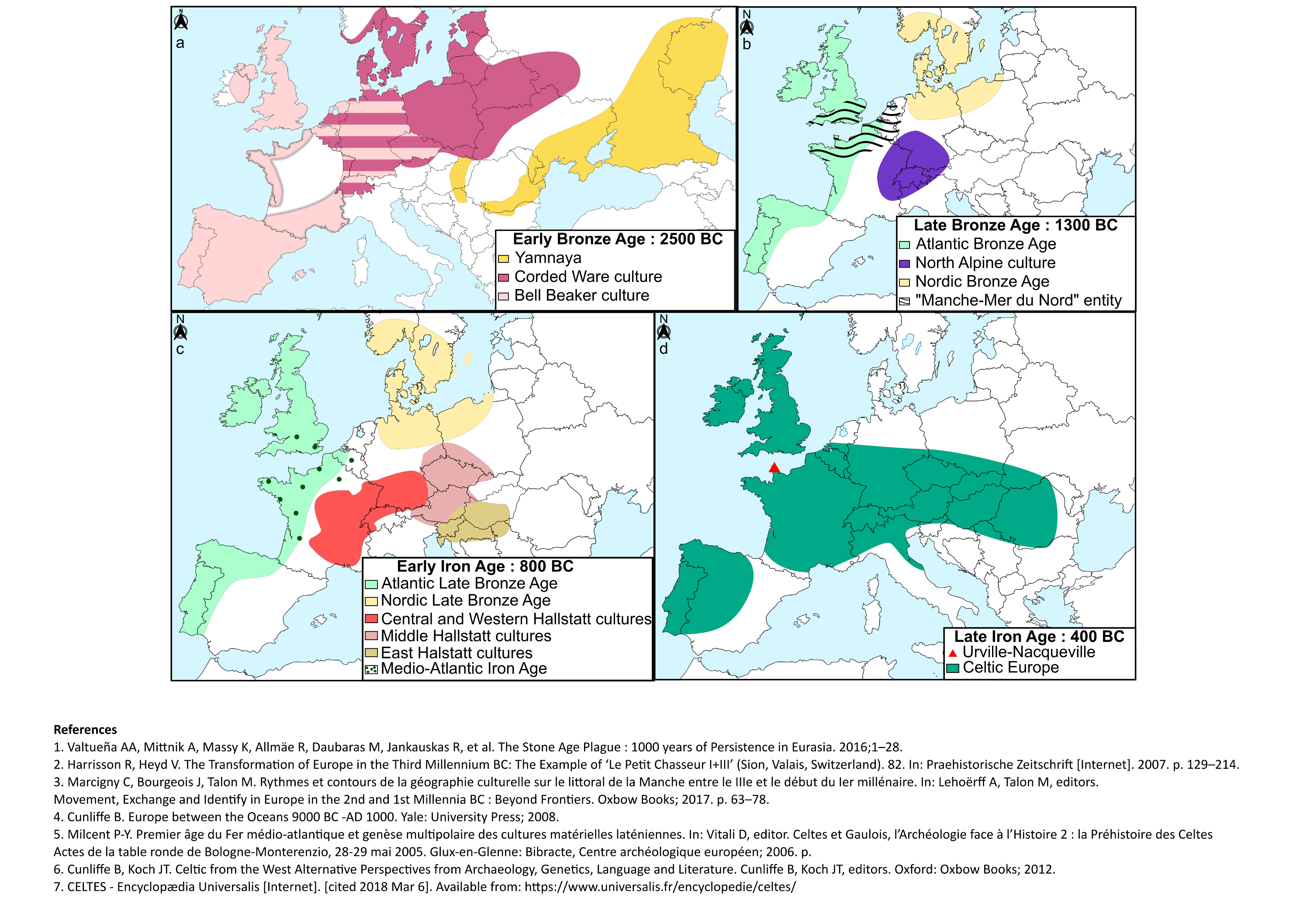

Supplement: S1 Fig — A) Late Neolithic / Early Bronze Age cultures, approximately 2300 BC (based on [SI.1] and [SI.2]). B) Late Bronze Age cultures and the “Manche-Mer du Nord complex”, approximately 1300 BC (based on [SI.3] and [SI.4]). C) Early Iron Age cultures and the Medio-Atlantic Iron Age, approximately 800 BC (based on [SI.5] and [SI.6]). D) Late Iron Age: celtophone Europe approximately 400 BC (map based on [SI.7]). (TIFF) [file pone.0207459.s001.tiff]

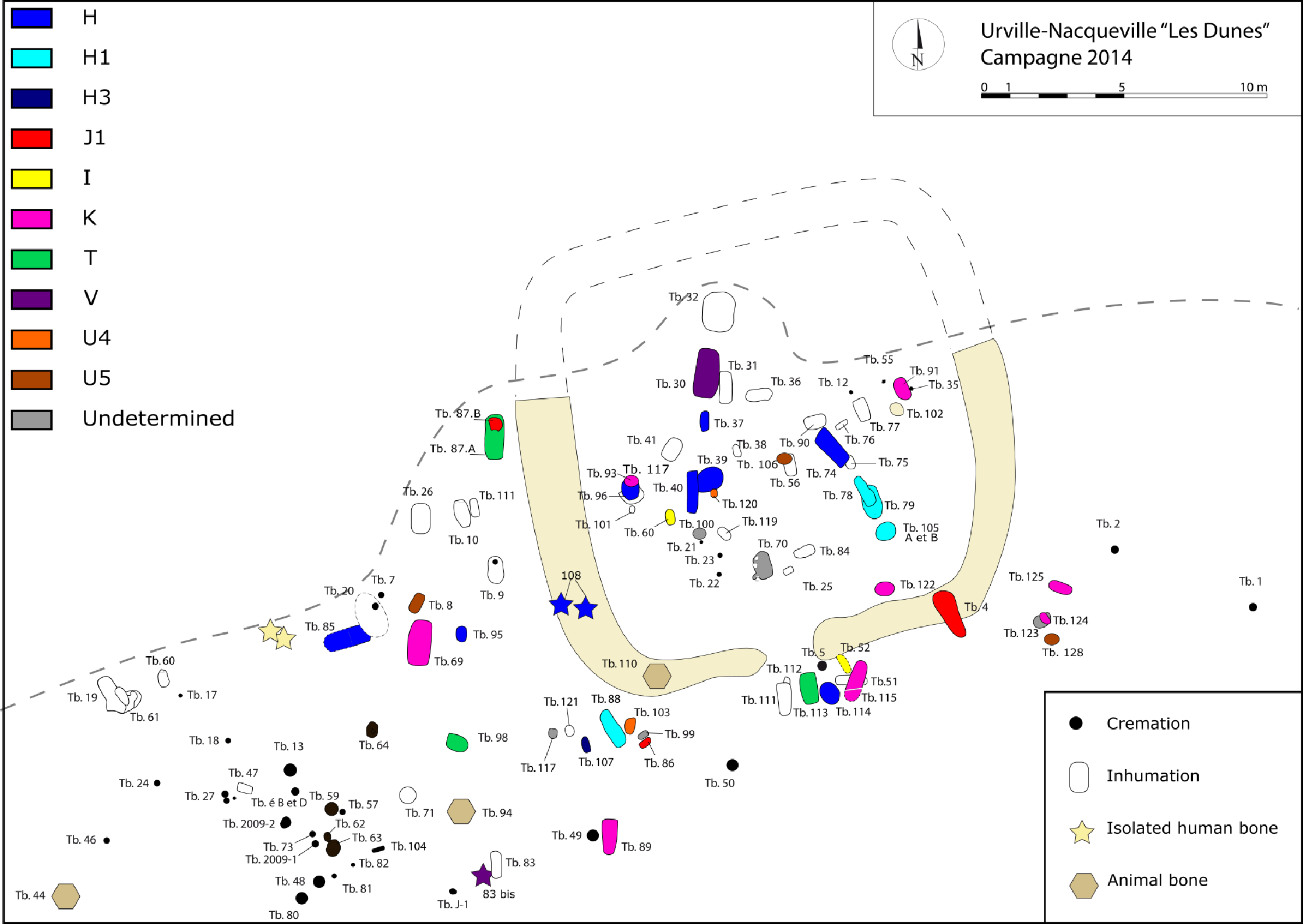

Supplement: S2 Fig — (TIFF) [file pone.0207459.s002.tiff]

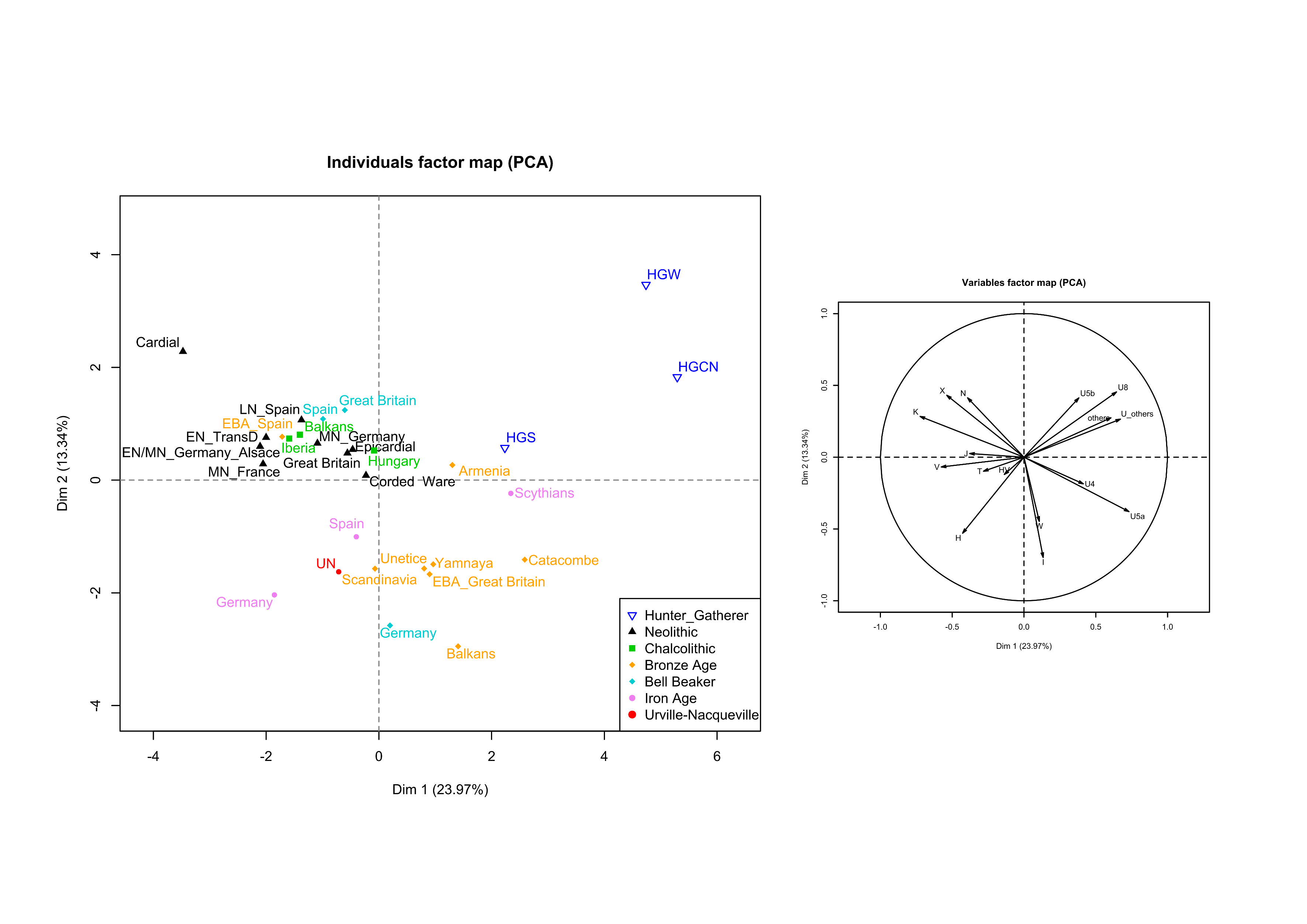

Supplement: S3 Fig — (TIFF) [file pone.0207459.s003.tiff]

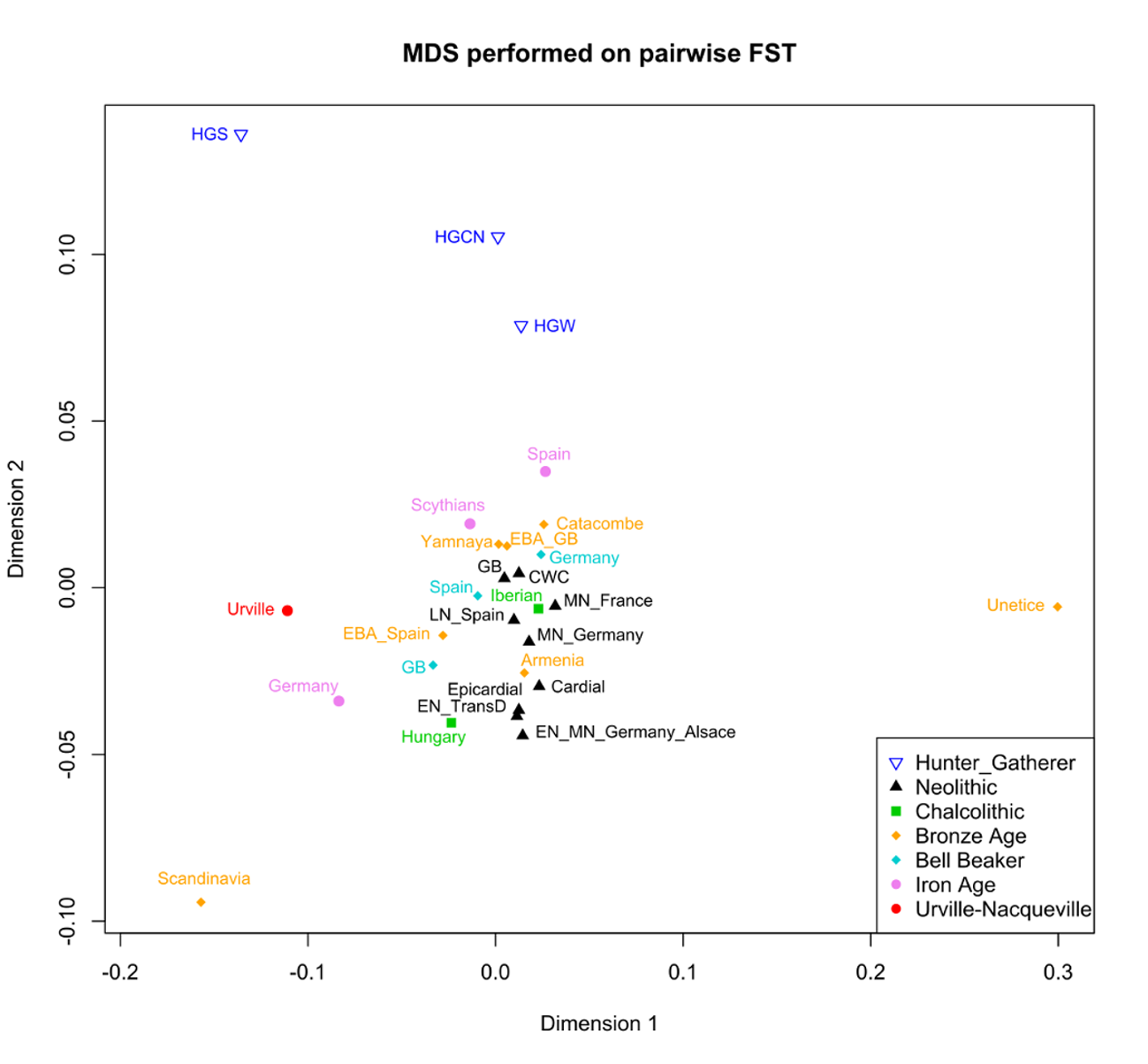

Supplement: S4 Fig — (TIFF) [file pone.0207459.s004.tiff]
