# Supplementary material for: The multiple maternal legacy of the Late Iron Age group of Urville-Nacqueville (France, Normandy) documents a long-standing genetic contact zone in northwestern France
Source: PLoS One. 2018 Dec 6;13(12):e0207459. doi: 10.1371/journal.pone.0207459 (PMC6283558; doi:10.1371/journal.pone.0207459)
Supplement: S1 File — (DOCX) [file pone.0207459.s017.docx]

**References for S1. figure**

SI.1. Valtueña AA, Mittnik A, Massy K, Allmäe R, Daubaras M, Jankauskas R, et al. The Stone Age Plague : 1000 years of Persistence in Eurasia. 2016;1–28.

SI.2. Harrisson R, Heyd V. The Transformation of Europe in the Third Millennium BC: The Example of ‘Le Petit Chasseur I+III’ (Sion, Valais, Switzerland). 82. In: Praehistorische Zeitschrift [Internet]. 2007. p. 129–214.

SI.3. Marcigny C, Bourgeois J, Talon M. Rythmes et contours de la géographie culturelle sur le littoral de la Manche entre le IIIe et le début du Ier millénaire. In: Lehoërff A, Talon M, editors. Movement, Exchange and Identify in Europe in the 2nd and 1st Millennia BC : Beyond Frontiers. Oxbow Books; 2017. p. 63–78.

SI.4. Cunliffe B. Europe between the Oceans 9000 BC -AD 1000. Yale: University Press; 2008.

SI.5. Milcent P-Y. Premier âge du Fer médio-atlantique et genèse multipolaire des cultures matérielles laténiennes. In: Vitali D, editor. Celtes et Gaulois, l’Archéologie face à l’Histoire 2 : la Préhistoire des Celtes Actes de la table ronde de Bologne-Monterenzio, 28-29 mai 2005. Glux-en-Glenne: Bibracte, Centre archéologique européen; 2006. p.

SI.6. Cunliffe B, Koch JT. Celtic from the West Alternative Perspectives from Archaeology, Genetics, Language and Literature. Cunliffe B, Koch JT, editors. Oxford: Oxbow Books; 2012.

SI.7. CELTES - Encyclopædia Universalis [Internet]. [cited 2018 Mar 6]. Available from: https://www.universalis.fr/encyclopedie/celtes/
